# Expanded Haemodialysis as a Current Strategy to Remove Uremic Toxins

**DOI:** 10.3390/toxins13060380

**Published:** 2021-05-26

**Authors:** Paola Ciceri, Mario Cozzolino

**Affiliations:** 1 Renal Research Laboratory, Department of Nephrology, Dialysis and Renal Transplant, Fondazione Ca’ Granda IRCCS, Ospedale Maggiore Policlinico, 20122 Milan, Italy; p.ciceri@hotmail.it; 2 Renal Division, ASST Santi Paolo e Carlo, Department of Health Sciences, University of Milan, 20142 Milan, Italy

**Keywords:** uremic toxins, chronic kidney disease, cardiovascular disease, vascular calcification, dialysis

## Abstract

Chronic kidney disease (CKD) is characterized by the retention of solutes named uremic toxins, which strongly associate with high morbidity and mortality. Mounting evidence suggests that targeting uremic toxins and/or their pathways may decrease the risk of cardiovascular disease in CKD patients. Dialysis therapies have been developed to improve removal of uremic toxins. Advances in our understanding of uremic retention solutes as well as improvements in dialysis membranes and techniques (HDx, Expanded Hemodialysis) will offer the opportunity to ameliorate clinical symptoms and outcomes, facilitate personalized and targeted dialysis treatment, and improve quality of life, morbidity and mortality.

## 1. Introduction

In the recent years, there have been many significant achievements in the management of end-stage renal disease (ESRD) patients on dialysis. The treatment of bone mineral metabolism, anaemia, electrolyte imbalance, and anticoagulation has progressed. Moreover, the handling of comorbidities, such as cardiovascular disease and diabetes, has improved [1]. In the last decades, new technologies related to haemodialysis and hemodialfiltration have been developed. Nevertheless, in the last 30 years there have not been displayed clear mortality advantages resulting from new dialytic techniques. In fact, despite these innovations, it is still needed to make the appropriate efforts to achieve further advance in the care of ESRD patients in order to ameliorate clinical outcomes. 

The most currently applied dialytic technique are conventional HD (haemodialysis) or HDF (online hemodiafiltration). There are many dialyzers utilized, generally sub-grouped in 4 classes: low flux (LF); high flux (HF); high cut-off (HCO) and medium cut-off (MCO). The combination between conventional HD and MCO dialyzer has been recently defined as ‘expanded hemodialysis’ (HDx).

Since there is still a significant difference in life expectation between general population and ESRD patients, in the last decade one of the central point that has been identified as a potential modifiable item is the efficient removal of uremic toxins by dialysis treatment. In fact, LF membranes can retain several uremic toxins, while, with the development of HF membranes, there was an improvement in their clearance. Interestingly, HF membranes are effectively able to remove uremic toxins in a range of small solutes (urea) and middle molecules (β2-microglobulin). However, the efficient removal of middle molecules (MM) uremic toxins, in a molecular range of 15–50 KDa, is currently limited. In this family of MM, 27 uremic toxins have been described, with a 1.5–200 folds increased circulating levels in ESRD patients. These uremic toxins have a large spectrum of chemical and functional variety and are roughly divided in 4 groups: cytokines, adipokines, immune-related proteins, and growth factors and hormones. Their increased pathological concentration in ESRD patients, due to kidney failure, seems to be responsible of decreased chemiotaxis and immune-defence, atherogenesis, cardiac hypertrophy, and anorexia, accounting for the MM detrimental roles in inflammation, calcification, and cardiovascular morbidity and mortality. Moreover, ESRD patients have an increased risk of mild cognitive impairment and dementia probably due to an accumulation of uremic neurotoxins in the CNS [2]. MM uremic toxins can be further divided in two sub-class: small middle molecules (SMM) 0.5–25 KDa and large middle molecules (LMM) >25 KDa. Clearly, HD with HF membranes demonstrated poor removal of LMM. On the contrary HDF at high volumes (>23 L/session) produced better results, although high blood flows are required. More recently, many efforts were put in trying to develop dialysis membranes that allow a better MM removal. Encouraging results has been obtained with HCO membranes, but albumin loss represented a limitation to their practical application. 

Recently, the unmet need to address clearance of uremic toxins has tried to be faced up with the development of MCO membranes. By a process of spinning the membrane polymer through a solvent, the hollow fiber hemodialysis membrane is obtained. The pores within these membranes are not uniform and have a “bell-shaped” distribution of size, from small to large, with the largest of these pores that is still smaller than albumin. In the MCO filters, the distribution of the pores within the dialysis membranes have been changed to a tighter distribution, compared to HCO filters, to allow for the removal of MM with molecular mass between 15 and 60 kDa, without excessive albumin loss. In fact, the higher number and the tighter size pore distribution, with more uniform pores, allowed for the same mean size pore of HCO, without pores big enough to allow a significant albumin removal. MCO-HD (HDx) indicates the dialytic technique in which diffusion and convection are combined inside a hollow fiber dialyser. The introduction of MCO membranes in clinical practice is one of the most relevant innovations in the field of haemodialysis. These filters are capable of removing MM uraemic toxins without the need for high convective volumes and without a significant albumin loss [3].

More recently, several studies have been published with the main aim to elucidate which MM can be efficiently removed by MCO and to assess the clinical relevance for dialysis patients. A great deal of attention has also been given to albumin loss and clinical signs of hypoalbuminuria following HDx. Here we review the literature on clinical HDx experience with a focus on uremic toxins removal efficiency, safety, patient quality of life (QOL) and outcomes. We analyze also the scientific research that contributes to elucidate the potential benefits given by the better removal of uremic toxins and their pathogenic effect in particular in the processes of inflammation and calcification in experimental models.

## 2. HDx Clinical Evidences

### 2.1. Uremic Toxin Removal Efficacy

#### 2.1.1. HDx vs. HDF 

The first important clinical question is: is there any difference between HDx and HDF? A first group of studies were focused on MM removal efficacy by HDx compared to HDF. It has been demonstrated that HDF allows a better removal of middle molecules in comparison to HD. Is this true also when HDF is compared to HDx? LMM removal is more dependent to convective than diffusive transport compared to small molecules removal. In general, LMM clearance is dependent to the product of sieving coefficient and convective volume. MCO membranes achieve convective transport by varying the pressure gradient over the filter, creating a very high back-filtration that ultimately gives a moderate convection volume. Thus, compared to conventional HD, HDx gives a better LMM removal due to a higher sieving coefficient and a moderately high internal convection volume [4]. Compared to HDF, which has a higher convection volume, HDx has given ambiguous results in MM clearance. Five studies have been published comparing pre-post dialysis efficacy in MM removal by HDx compared with HDF (Table 1). Three studies demonstrated a comparable reduction rate (RR) for all the MM measured [5,6,7], whereas two other studies demonstrate better RR by HDx for some MM [8,9]. MM uremic toxins that may be better removed by HDx are: myoglobin, β-2 microglobulin, complement factor D (CFD), α-1 microglobulin, κ and λ free light chains (FLC) and YKL40. No all data go in the same direction, since the same toxins in other studies have a similar RR, i.e., myoglobin and β-2 microglobulin [5]. Moreover, a long-term study by Belmouaz [5] failed to show differences between HDx and HDF. One explanation to these different results might be the difference in convection volumes utilized in the studies that makes difficult to compare the different results. Another limitation is the small number of patients analyzed. Nevertheless, from these clinical studies on HDx uremic toxin removal, it seems that MCO filters have at least an equal MM clearance efficacy compared to HDF. Final answer will come from future studies, in particular from larger trial analysis of LMM in the molecular weight range of 40–60 KDa, since LMM should be better removed by HDx compared to HDF.

#### 2.1.2. HDx vs. Conventional HD

Recently, data have been published comparing the efficiency in MM removal between MCO and HD, in a randomized clinical trial [14]. Generally, other studies are non-randomized clinical trials with a limited number of patients. Two are the parameters evaluated, the removal of MM uremic toxins pre and post a dialytic session and long-term clearance (Table 1). RR intra-dialytic session accounts for the efficacy of the membrane in removing MM uremic toxins, but long-term studies, that evaluate pre-dialysis levels of MM, are crucial because if there is toxicity associated with retained uremic solutes, therapeutic management will require their sustained reduction. Out of 11 studies that evaluated the difference between HF and MCO in MM removal, only two in pre-post dialysis sessions [12,13] and another one in long term evaluation [11] reported lack to find any difference. In contrast, the majority of performed studies reported a significant increased MM removal by MCO (Table 1). MM uremic toxins evaluated are both in the range of SMM (up to 25 KDa) and LMM (from 25 to 50 KDa) (Table 2). Almost every study analyzed a miscellaneous of SMM and LMM demonstrating a greater removal by MCO compared to HD both in the range of SMM and LMM where, due to the filter characteristic, a better performance was expected (Table 1).

Two recent trials demonstrated a reduction in the concentration of FLC [15,20]. One of the studies was similar in design to ours [3]: a randomized, open-label, cross-over study, with 40 patients who were divided to carry out 3 months of MCO-HD followed by 3 months of HF-HD, or vice versa [20]. The primary outcome was myoglobin (17 kDa) RR after 3 months of MCO-HD compared to HF-HD. The RR for any given solute equals the subtraction of the pre-session from the post-session concentration, divided by the pre-session concentration. A significant increase in myoglobin RR was found during the MCO dialyzer intervention period, even if the pre-dialysis myoglobin concentration was similar in both groups, probably due to a rebound effect and redistribution of this protein into the blood compartment. Secondary outcomes included RR and pre-dialysis serum concentration of other middle molecules such as β2-microglobulin (11.8 kDa), tumor necrosis factor-alpha (17.2 kDa), IL-6 (21 kDa), kappa FLC (22 kDa), prolactin (23 kDa), and lambda FLC (45 kDa). Importantly, not only was the RR of β2-microglobulin and FLC higher with the MCO dialyzer, but the pre-dialysis serum concentrations of β2-microglobulin and FLC were also lower during the MCO-HD phase, demonstrating that the effects of higher removal of MM uremic toxins were sustained until the following dialysis session. Accordingly, there was no difference in RR and pre-dialysis serum concentration for tumor necrosis factor-alpha (17.2 kDa) and IL-6 (21 kDa), independently by dialysis membrane.

These MM uremic toxins have been predominantly involved in inflammation, immune-response and cardiovascular disease in ESRD (Table 2). Nevertheless, there are not sufficient data from randomized control trials that demonstrate an effect of the MM improved clearance by MCO on clinical outcomes. However, all the study on HDx demonstrate its real efficacy in MM clearance, considering RR values indicative for surrogate markers of potential clinical benefits.

Other studies evaluated the effect of HDx on the removal of medications or coagulation and erythropoiesis stimulating agents (ESA). Allawati et al. [46], in a pharmacokinetic study on 5 patients, found that vancomycin clearance is not different when MCO filters are utilized compared to HF. In an in vitro study [47], filtered plasma was spiked with vancomycin, erythropoietin (EPO), heparin, coagulation factors and insulin to compare the retention between MCO vs. both HF and HDF. In this experimental setting, no changes of any medication dosing were necessary after the switch from either HD or HDF to MCO, probably due to the fact that the increase in pore size does not enhance MCO filter permeability. This was also demonstrated in this study for the coagulation factors analysed that have a molecular weight in the range of albumin. The relationship between MM uremic toxins removal and medication has also been investigated by Lim et al. [48] that demonstrated in 49 patients how HDx reduces ESA resistance compared to HD. It has been hypothesized a link between a better TNF-α removal found with MCO and a possible consequent reduced ESA resistance, likely associated with a lower inflammatory state. Thus, MCO seems to improve iron metabolism, and the authors demonstrate that this effect is independent by hepcidin. Nevertheless, no effect on ESA dose was found in other two studies [17,19]. The general oxidative stress and inflammatory status of patients dialyzed by MCO compared with LF and HF was also evaluated [49]. An effect of inflammation improvement, evaluating C-reactive protein removal, was investigated, with no effect of MCO on oxidative stress biomarkers. Recently, we investigated protein bound uremic toxins (PBUT) levels in a prospective, open-label, controlled, cross-over pilot study comparing HDx and HD [50]. Interestingly, a significant decrease in CMPF (3-Carboxy-4-methyl-5-propyl-2-furanpropionate), tryptophane and some of its metabolites such as indoxyl sulphate, 3 indol acetic acid, kynurenine was found in the HDx group. Since the PBUT cannot be filtered, for the high molecular weight due to the binding with albumin, we hypothesized that the decrease might be due to the fraction of albumin loss that occurs during HDx. This hypothesis will imply that a minimal albumin loss, without any clinical symptoms of hypoalbuminaemia, might be beneficial, allowing a decrease in PBTU levels that have been difficult to eliminate with extracorporeal strategies until now. In fact, PBUTs cause various negative effects in ESRD patients, because their removal by conventional HD is severely limited by their low free fraction in plasma. Therefore, the concept that the dialytic removal of PBUTs should be increased is a suggestive hypothesis that needs deep investigation in larger clinical trials to be confirmed

## 3. Safety

### 3.1. Albumin Removal

The rationale of MCO membrane development has been the improvement of the LMM uremic toxins clearance, with a molecular weight close to albumin without removing a significant amount of albumin itself. This was achieved by increasing pore size and, at the same time, reducing pore size distribution compared to HF membranes. The previous experience with HCO filters, that were affected by a significant albumin removal, lead to a careful analysis of albumin retention by MCO by clinicians. In fact, serum albumin concentration is widely considered as a surrogate of health and good prognosis reflecting nutritional status and inflammatory burden in this population [51]. Thus, in every study, that analyzed HDx compared with either HD or HDF, albumin clearance was evaluated (Table 3). HDx resulted without a significant decrease of albumin in 12 out of the total 19 studies performed, and with a significant removal in 6 studies although it was not registered any clinical signs of hypoalbuminemia. The mixed results are independent by the duration of the study and overall they design HDx as a safe dialysis therapy able to keep serum albumin steady.

### 3.2. Microbial Contamination

The increased pore size of MCO membranes arises the question about a hypothetical increased permeability for endotoxins and other bacterial contaminants potentially present in dialysis fluid. The concerns are about an eventual chronic exposure to inflammatory products, such as cytokines, that can be induced by the increased permeability for bacterial degradation products, thus potentially contributing to the micro-inflammatory status of ESRD patients. Two studies have been performed to assess MCO safety related to microbial contamination using in vitro dialysis system [53,54]. Both of them compared LF, HF, MCO and HCO membranes. Schepers et al. added a filtered solution of lysate from two water-borne bacteria, *Pseudomonas Aeruginosa* and *Pseudomonas Saccharophila*, to the dialysate flow. They demonstrated that none of the membranes tested, included MCO, were able to allow translocation of bacterial products, even if the endotoxin level was at least 4 times higher than the upper limit of endotoxin load allowed. The same result was found by Hulko et al., adding *E. Coli* endotoxin and *Pseudomonas Aeruginosa* extract and detecting no crossing from dialysate to the blood side with all the membranes tested included the MCO filter. In conclusion, it appears that MCO membranes are safe and retain endotoxins as the other membranes, being suitable for haemodialysis using high-standard dialysis fluid quality.

### 3.3. Adverse Events

Both adverse events (AE) and serious AE (SAE) were evaluated in several studies in order to report the safety profile of MCO membranes. No dialysis reactions related to the use of the membrane were reported [3,8,10,14,15,27]. No significant differences were found in the number of either AE or SAE between the MCO and the other filters analyzed, and no AE related to albumin decrease were present [7]. Interestingly, we reported that the number of infections was lower in patients treated with HDx compared with HD, whereas there was no difference in the number of hospitalization and hypotension occurrence in the two groups [3]. However, this observation needs to be confirmed in larger randomized clinical trials. Taken together all the observations in different groups of patients treated with MCO lead to the conclusion that the MCO filters are safe and well-tolerated. 

## 4. Quality of Life (QOL)

Dialysis patients suffer from symptoms such as generalized weakness, fatigue, and pruritus probably related to an insufficient removal of uremic toxins by dialysis. Thus, these patients are affected by poor health-related QOL due to both uremic symptoms and dialysis treatment. In order to verify whether the improved clearance of MM with MCO might have a positive effect on patient-reported outcomes, some clinical studies were recently performed. Until now, 5 studies have been conducted comparing QOL in HDx compared with HD (Table 4). In 2 studies there were no differences in the self-reported patient outcomes [13,14], whereas in 3 others an improvement in the physical domains of QOL and uremic pruritus [12], in the Restless Legs Score (RLS) and an association with higher health-related quality of life scores [55], and in KDQoL-SF36 survey [16] were reported. Except for the study by Alarcon et al., all others measured the MM removal, finding a significant clearance of some MM with HDx, but a relationship between the better MM removal and change in QOL still need a deep evaluation and adequate powered clinical trials. Analyzing and trying to find strategies to preserve QOL is relevant for ESRD patients, since it has been demonstrated that both the decrease in mental and physical component score is associated with an increase in the risk of mortality [56]. New strategies should be developed in the future, to establish if HDx has an impact on QOL and to verify whether better MM uremic toxins removal might influence QOL and ultimately life expectancy. Therefore, it is important that dialysis prescriptions would be tailored to improve symptoms and quality of life based upon removal patterns of uremic solutes linked to outcomes. 

## 5. Potential Mechanisms and Pathogenetic Hypothesis 

The uremic milieu is a complex miscellaneous of different uremic toxins responsible for the increased mortality and morbidity in ESRD patients. Uremic toxins are a plethora of molecules characterized by different structures and biological activities. Uremic patient clinical picture is characterized by an accelerated vascular aging and by worse cardiovascular outcomes compared with the general population. Some studies have been performed to investigate whether HDx reduces inflammation or pro-calcific serum potential compared to conventional HD. Regarding chronic inflammation, ESRD is characterized by the retention of many inflammatory mediators that increase the chronic inflammatory state of the patient. Among the middle molecules with a molecular weight in the range of 15–50 KDa there are some with a pro-inflammatory profile, such as IL-1β, IL-6, TNFalpha, IL-18, soluble TNF receptor 1 (sTNF-R1), sTNF-R2, Pentraxin-3 and YKL-40. Two are the main points that may help in trying to elucidate the potential beneficial effect of HDx: (1) whether there is a better clearance of pro-inflammatory molecules with a decreased accumulation (see Removal efficacy section) and (2) whether the better filtration of these molecules during time may modify the inflammatory general state of the patient. To try to elucidate these aspects, especially the second one, two different studies have been performed recently. In 2017 Zickler et al. [10] demonstrated, in a randomized cross-over trial in 48 uremic patients, a better removal by MCO, compared to HD, of some inflammatory biomarkers (Table 1). They evaluated also, as primary end-point, the effect of HDx on peripheral leukocyte IL-6 and TNFalpha mRNA levels after 4 weeks of dialysis, finding a significant decrease induced by HDx. This result supports the hypothesis that HDx is able to ameliorate the inflammatory state of the patient. In fact, peripheral leukocyte IL-6 and TNFα mRNA levels are intracellular markers that are not affected by dialysis and demonstrate a decreased synthesis of these two mediators. Nevertheless, it is still unclear whether the significant modified serum levels of inflammatory molecules found in this study are due either to a better removal or to a decreased synthesis, or it is due to both the processes. In another study [57], the same authors investigated the renin-angiotensin-aldosterone system (RAAS) that is involved in the modulation of the local immune response by influencing the recruitment of circulating leukocytes to the site of inflammation. RAAS over-activation, therefore, can promote atherosclerotic plaque development, aggravating increased cardiovascular risk in uraemia. Interestingly, it has been observed a modification by HDx of the general status of the patient, with a decreased expression of ACE and ACE2 mRNA in leukocytes. The effect on RAAS is not direct, likely due to a better removal of some MM uremic toxins. Furthermore, the modulation of RAAS may contribute to a potential protective role of HDx on inflammation and atherosclerosis. 

There is a link between inflammation and calcification in uremia [58], since the increase in micro-inflammation in CKD patients can exacerbate vascular calcification [59]. Recently, an in vitro model of miniature dialysis [60] has been utilized to elucidate the efficacy of MCO dialysis membranes on removal of pro-inflammatory molecules and its influence on plasma pro-calcifying potential. Cytokine-rich plasma was obtained from healthy donor blood spiked with endotoxin. Removing pro-inflammatory middle molecules by MCO resulted in a less pro-calcifying potential of cytokine-rich plasma in a model of vascular smooth muscle cell (VSMC) calcification. Moreover, the cytokine-rich plasma MCO filtered was able to modulate the profile of released protein by VSMCs during calcification. This in vitro study emphasized the positive effect of MCO in removing pro-inflammatory molecules that in turn decrease calcification plasma potential, although the cytokine concentration in this experimental model was extremely high compared with the in vivo situation. In another study [61] it has been evaluated the effect of HDx compared with HD in 48 uremic patients, studying sera pro-calcifying effect in a model of VSMC calcification. MCO filtered sera had a decreased pro-calcifying effect, quantified by calcium deposition and alkaline phosphatase (ALP) activity after 4 and 12 weeks. In the MCO group there was also a decreased release of calcification-associated proteins such as MGP, OPN and GDF-15, probably due to a compensative mechanism following calcification inhibition. Apoptosis is also significantly decreased in the MCO group. This is a relevant data, since it has been demonstrated an involvement of apoptosis in the exacerbation of VSMC calcification [62,63].

**Figure 1:**
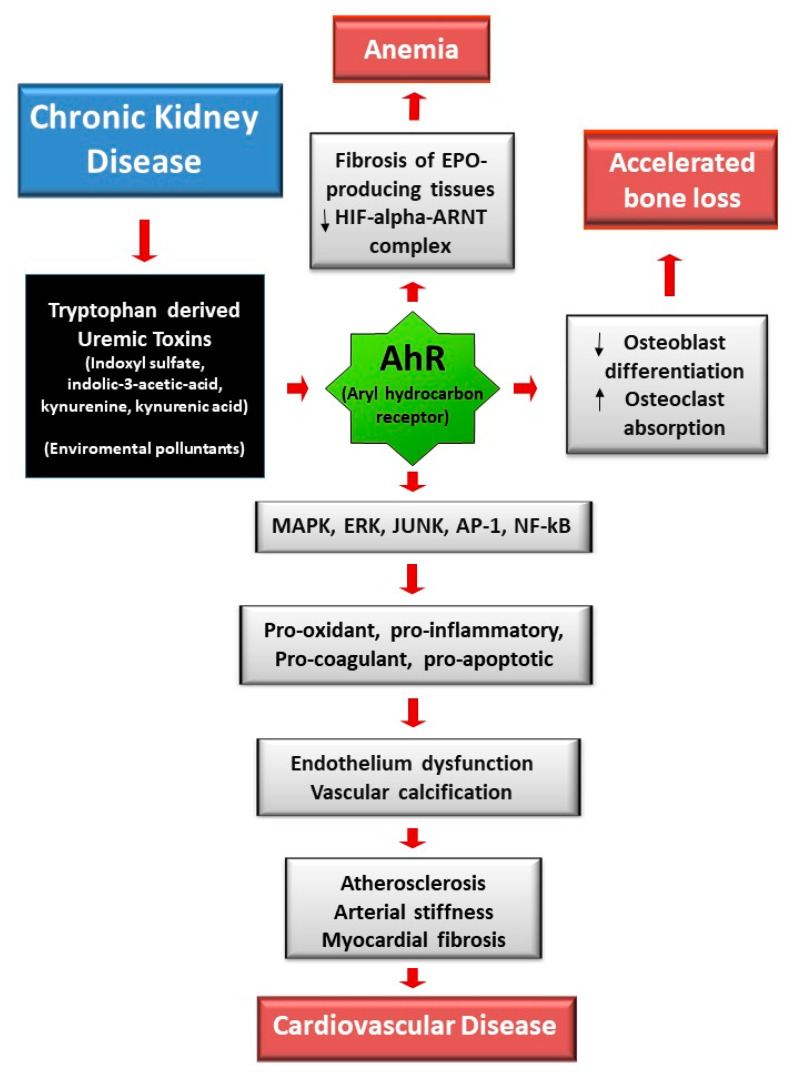
In uremia tryptophan derived uremic toxins are involved in ESRD comorbidities by binding to the Table 1. (Activator Protein 1); NF-kB (Nuclear Factor Kappa light chain—enhancer of activated B cells).

Recently, we reported a positive effect of HDx in decreasing pro-calcifying activity of uremic serum [24]. This study was a prospective, open-label, controlled, cross-over pilot study comparing HDx and conventional HD in 20 prevalent HD patients. The pro-calcifying effect of uremic serum was evaluated in a model of high-phosphate (high-Pi) induced calcification in VSMCs finding a less pro-calcifying potential from HDx-treated patient sera compared to HD. The analysis of the pathogenic processes involved in high-Pi induced calcium deposition showed that uremic serum of HDx-treated patients induced less VSMC necrosis compared to uremic serum of HD patients, with no significant effect on apoptosis and VSMC osteogenic differentiation. Some protein bound uremic toxins have been analyzed in patient sera, finding that CMPF, tryptophane and some of its metabolites, are better removed by HDx [50]. This is an interesting result since uremic toxins derived from tryptophan metabolism have been associated with cardiovascular diseases in CKD patients. In fact, uremic toxins derived by the tryptophan metabolism are endogenous ligands of the transcription factor aryl hydrocarbon receptor (AhR) which is involved in cardio- toxicity and vascular inflammation (Figure 1). Moreover, AhR activation mediates pro-oxidant, pro-inflammatory, pro-coagulant and pro-apoptotic effects on cells of the cardiovascular system [64].

## 6. Conclusions

During the last five years, advances in understanding of uremic toxins as well as the availability of new dialysis membranes and techniques has led to improve removal of uremic retention solutes. The most recent and promising advance in the field of haemodialysis is represented by the development of medium-cut-off (MCO) high-retention-onset membranes. Expanded haemodialysis (HDx) has been introduced to describe a dialysis modality in which diffusion and convection are combined inside a hollow-fibre dialyser containing an MCO membrane. MCO membranes are capable of removing medium-high uremic toxins without the need for high convective volumes and without a significant albumin loss. Since uremic toxins have been linked to the development of the chronic inflammatory state typical of ESRD and dialysis, to the formation of vascular calcification and, consequently, to the incidence and severity of adverse cardiovascular events, the implementation of a dialysis technique able to better clear these molecules may have a major impact on the outcomes of haemodialysis patients.

## Figures and Tables

**Table 1:** Effect of MCO membranes on uremic toxin removal efficacy.

	Year	1st Author Publication	N Patients	Dialysis Treatment	Time	Study Design	MM Significantly Removed by MCO Pre- and Post-Dialysis	MM Significantly Removed by MCO End Study
**HDx vs. HDF**	2017	Kirsch [8]	39	HDF+HF HD+HF HD+MCO		One midweek dialysis	Myoglobin, beta-2 microglobulin, kappa and lambdaFLC, CFD, alpha1-microglobulin, YKL40 *	-
	2018	Belmouaz [5]	10	HDF+HF HD+MCO	12 months	Switch from OL-HDF to HD MCO	-	No difference
	2018	Garcia-Prieto [6]	18	HDF+HF HD+HF HD+MCO	3 weeks	Midweek dialysis, 3 consecutive weeks	Myoglobin, beta-2 microglobulin, prolactin, alpha1 acid glycoprotein **.	-
	2019	Kim [9]	6	HDF+HF HD+HF HD+MCO	3 weeks	Midweek dialysis, 3 consecutive weeks	Myoglobin, lambda FLC *	-
	2019	Maduell [7]	22	HD+MCO+ HDF+8 different dialyzers	9 weeks	9 dialysis sessions once a week	No difference	-
**HDx vs. HD**	2017	Zickler [10]	48	HD, MCO vs. HF	12 weeks	12 weeks (4+8)	-	kappa and lambda FLC, sTNFR1
	2019	Cho [11]	57	HD, MCO vs. HF	12 months	12 months	beta-2 microglobulin, kappa and lambda FLC, CFH	No difference
	2020	Lindgren [12]	16	HDF+HF HD+MCO	4 weeks	1 single dialysis section OL-HDF-2w washout-MCO	No difference	-
	2020	Lim [13]	49	HD, MCO vs. HF	12 weeks	12 weeks	No difference	kappa and lambda FLC
	2020	Weiner [14]	172	HD, MCO vs. HF	24 weeks	24 weeks	-	beta-2 microglobulin, CFD, kappa and lambda FLC, TNFalpha
	2020	Krishnasamy [15]	89	HD, MCO vs. HF	32 w	4 weeks HF+24 week MCO +4 weeks HF	-	kappa and lambda FLC
	2020	Sevinc [16]	52	HD, MCO vs. HF	6 months	3 months+3 months pre-post dialysis	Myoglobin, beta-2 microglobulin, kappa and lambda FLC (RR)	beta-2 microglobulin, kappa and lambda FLC, VEGF
	2020	Reis [17]	15	HD, MCO vs. HF	2 m	5 sessions/week 2 h 30 min each (short frequent HD)	-	Prolactin
	2020	Perez-Alba [18]	7	HD, MCO	12 months	Home hemodialysis	-	beta-2 microglobulin
	2020	Rambabova [19]	4	HD, MCO vs. HF	12 weeks	Pre-post dialysis	Myoglobin, beta-2 microglobulin, kappa and lambda FLC	-
	2020	Belmouaz [20]	40	HD, MCO vs. HF	6 m	3 months+3 months pre-post dialysis	beta-2 microglobulin, kappa and lambda FLC	Myoglobin, beta-2 microglobulin, prolactin, FGF23, homocysteine, kappa and lambda FLC (RR)

* compared to both HD-HF and OL-HDF. ** compared to HD-HF. no differences with OL-HDF, MM: middle molecules; HD: haemodialysis; HDF: oneline hemodiafiltration; HDx: hexpanded haemodialysis; MCO: medium cut-off membrane; HF: high flux membrane; FLC: free light chain; CFD: complement factor D; CFH: cell-free hemoglobin; TNF: tumor necrosis factor; sTNFR: soluble tumor necrosis factor receptor; VEGF: vascular endothelial growth factor; -: not measured.

**Table 2:** Middle molecules pathological involvement in uremia.

	Middle Molecule.	MW (KDa)	Possible Role in Uremia	Studies Evaluating the Removal
**SMM**	beta2 microglobulin	11	Dialysis related amyloidosis, inflammation, immune-dysfunction, mortality [21,22]	[6,7,8,11,12,14,16,18,19,20]
	myoglobin	16.7	Oxidative stress, mitochondrial dysfunction, organ damage [23,24,25]	[6,7,8,9,12,16,19]
	TNFalpha	17.3	Left ventricular hypertrophy, anorexia, protein muscle breakdown [26,27,28]	[14]
	prolactin	22	Cardiovascular events, amplification of inflammatory cytokine response [29,30]	[6,7,17,20]
	kappa FLC	22.5	Inflammation, infection, mortality [31]	[8,10,11,13,14,15,16,19,20]
	FGF-23	22.5	Cardiovascular events [32,33]	[20]
	CFD	24	Over-activity of complement system [34]	[8,14]
	beta trace	25	Atherosclerosis, cardiovascular mortality [35,36,37]	[12]
**LMM**	alpha1 microglobulin	26	Inhibition leukocyte migration, chemiotaxis, IL-2 secretion [38,39]	[7,8]
	sTNFR1	34	Cardiovascular events [26,40]	[10]
	troponin T	35	Cardiovascular events [41,42]	[12]
	YKL40	40	Local tissue inflammation and fibrosis [38,43]	[8]
	alpha1 acid glycoprotein	41	Inhibition leukocyte migration, secondary immunodeficiency [44]	[6,7]
	VEGF	42	Cardiomyopathy, left ventricular dysfunction [45]	[16]
	lambda FLC	45	Inflammation, infection, mortality [31]	[8,9,10,11,13,14,15,16,19,20]

SMM: small middle molecules; LMM: large middle molecules; MW: molecular weight; CFD: complement factor D; FLC: free light chain; TNF: tumor necrosis factor; sTNFR: soluble tumor necrosis factor receptor; VEGF: vascular endothelial growth factor.

**Table 3:** Effect of MCO membranes on albumin removal.

	Year	1st Author Publication	N pz	Dialysis Treatment	Time	Study Design	Albumin Significant Reduction by MCO	Albumin Values (Baseline vs. End of MCO Period)
**HDx vs. HDF**	2017	Kirsch [8]	39	HD+HF HDF+HF HD+MCO		One midweek dialysis	tendency *	2.9–3.2 g filter AA 4.8–4.9 g filterBB 7.3 gr filter CC **
	2018	Belmouaz [5]	10	HD, MCO	12 months	Switch from OL-HDF to HD MCO	No	
	2018	Garcia-Prieto [6]	18	HDF+HF HD+HF HD+MCO	3 weeks	Midweek dialysis, 3 consecutive weeks	No	0.03 ± 0.01 g/session
	2019	Kim [9]	6	HD+HF HDF+HF HD+MCO	3 weeks	Midweek dialysis, 3 consecutive weeks	No	3.77 ± 0.3 to 3.58 ± 0.32 g/dL pre-post dialysis session
	2019	Maduell [7]	22	HD+MCO HDF with 8 different dialyzers	9 weeks	9 dialysis sessions once a week	No	10.3 ± 6.5 RR% pre-post dialysis session
**HDx vs. HD**	2017	Zickler [10]	48	HD, MCO vs. HF	12 weeks	12 weeks (4 + 8 extension)	Yes	37.0 ± 3.6 to 35.3 ± 3.7 g/L
	2019	Cozzolino [3]	20	HD, MCO vs. HF	6 months	3 months+3 months	Yes	−0.45 g/dL
	2019	Cho [11]	57	HD, MCO vs. HF	12 months	12 months	No	3.96 ± 0.31 to 3.94 ± 0.37 g/dL
	2020	Lindgren [12]	16	HDF+HF HD+MCO	4 weeks	1 single dialysis section OL-HDF-2w wo-MCO	No	−2.02 ± 3.9 RR% pre-post dialysis session
	2020	Lim [13]	49	HD, MCO vs. HF	12 weeks	12 weeks	No	4.11 ± 0.38 vs. 3.98 ± 0.27 g/dl
	2020	Weiner [14]	172	HD, MCO vs. HF	24 week	24 week	No	4.0 ± 0.3 vs. 4.0 ± 0.3 g/dl
	2020	Krishnasamy [15]	89	HD, MCO vs. HF	32 weeks	4 weeks HF + 24 weeks MCO + 4 weeks HF	No	35.8 ± 3.9 vs. 35.1 ± 4.0 g/L
	2020	Sevinc [16]	52	HD, MCO vs. HF	6 months	3 months+3 months pre-post dialysis	Yes	3.88 to 3.62 g/L
	2020	Reis [17]	15	HD, MCO vs. HF	2 months	5 sessions/week 2 h 30 min each	Yes	39.9 ± 3.7 vs. 38.3 ± 3.3 g/L
	2020	Perez-Alba [18]	7	HD, MCO	12 months	Home haemodialysis	No	
	2020	Rambabova [19]	4	HD, MCO vs. HF	12 weeks	Pre-post dialysis	No	40.50 ± 4.79 vs. 42.25 ± 4.50 g/L
	2020	Belmouaz [20]	40	HD, MCO vs. HF	6 months	3 months+3 months pre-post dialysis	Yes	38.2 ± 4.1 vs. 36.9 ± 4.3 g/L
	2020	Yeter [24]	42	HD, MCO; HF; LF	6 months	6 months	No	4.00 ± 0.25 vs. 3.84 ± 0.26 g/dl
	2020	Bunch [52]	638	HD, MCO	12 months	12 months	Yes	−1.8% cumulative change

HD: haemodialysis; HDF: oneline hemodiafiltration; HDx: hexpanded haemodialysis; MCO: medium cut-off membrane; HF: high flux membrane; AA Theranova 400 dialyzer, BB and CC prototype MCO dialyzers [4]. * no statistic is provided; ** median values.

**Table 4:** Effect of MCO membranes on uremic patient quality of life.

Year	1st Author Publication	N pz	Dialysis Treatment	Time	Study Design	Parameter Tested	Parameters Significantly Improved by MCO
2020	Lim [13]	49	HD, MCO vs. HF	12 weeks	12 weeks	KDQoL-SF36, pruritus	Physical functioning, physical role, morning pruritus distribution, frequencing of scracing during sleep.
2020	Weiner [14]	172	HD, MCO vs. HF	24 weeks	24 weeks	KDQoL-SF36, EQ-5D-5L	No difference
2020	Krishnasamy [15]	89	HD, MCO vs. HF	32 weeks	4 weeks HF + 24 weeks MCO + 4 weeks HF	6 monthsWT, MIS, RLS, QOL	No difference
2020	Reis [17]	15	HD, MCO vs. HF	2 months	5 sessions/week 2 h 30 min each (short frequent HD)	KDQoL-SF36	KDQoL-SF36
2020	Alarcon [54]	638	HD, MCO vs. HF	12 months	12 months	KDQoL-SF36, DSI, RLS	RLS 3 KDQoL-SF36 domains: -symptoms -effects of kidney disease -burden of kidney disease

KDQoL-SF36: Kidney Disease Quality of Life 36-Item Short Form Survey; DSI: Dialysis Symptom Index; RLS: Restless Legs Score; 6-min Walk Test; MIS: Malnutrition Inflammation Score; QOL: Quality of Life; EQ-5D-5L: EuroQol Instrument. RLS: Restless legs syndrome; 6 monthsWT: 6-min walk test; MIS: Malnutrition inflammation score.
